# Recent Advances in Nanotherapeutics for Multiple Myeloma

**DOI:** 10.3390/cancers12113144

**Published:** 2020-10-27

**Authors:** Daniela Iannazzo, Roberta Ettari, Salvatore Giofrè, Ali H. Eid, Alessandra Bitto

**Affiliations:** 1 Department of Engineering, University of Messina, 98166 Messina, Italy; diannazzo@unime.it; 2 Department of Chemical, Biological, Pharmaceutical and Environmental Chemistry, University of Messina, 98165 Messina, Italy; rettari@unime.it (R.E.); sgiofre@unime.it (S.G.); 3 Department of Basic Medical Sciences, College of Medicine, QU Health, Qatar University, 2713 Doha, Qatar; ae81@aub.edu.lb; 4 Biomedical and Pharmaceutical Research Unit, QU Health, Qatar University, 2713 Doha, Qatar; 5 Department of Pharmacology and Toxicology, Faculty of Medicine, American University of Beirut, 11-0236 Beirut, Lebanon; 6 Department of Clinical and Experimental Medicine, University of Messina, 98125 Messina, Italy

**Keywords:** multiple myeloma, liposome, nanotherapeutics, nanotechnology

## Abstract

**Simple Summary:**

Nanotherapeutics are useful tools to improve the deliverability of drugs, especially anti-cancer drugs that need to target specific cells. Several approaches have been studied for multiple myeloma, considering that immune cells are not easy to target with the available drugs. These pharmacological agents are administered in various combinations using Thalidomide (or Lenalidomide, Pomalidomide), corticosteroids (Dexamethasone), proteasome inhibitors (Bortezomib, Carfilzomib, Ixazomib), deacetylase inhibitors (Panobinostat), and monoclonal antibodies (Elotuzumab, Daratumumab). As all drugs these agents might have serious side effects and in addition, the reliance on stochastic events to deliver drugs to tumors reduces their effectiveness either through rapid clearance from blood or inadequate concentration in cancer cells. To address these issues liposomes, micelles, polymeric nanoparticles, inorganic nanoparticles, and carbon-based nanomaterials have been successfully tested in vivo and can be considered as useful tools to improve delivery of active pharmaceuticals that show poor bioavailability or poor internalization into myeloma cells.

**Abstract:**

Anticancer therapies cannot be included in a one-size-fits-all scenario; it is imperative to adapt therapies to the tumor molecular profile and most importantly to develop target-specific therapeutics. Nanotherapeutics can combine molecular imaging with molecular therapy in order to provide the maximum benefit to patients in terms of disease prevention, identification, and treatment. Nanotechnology applied to therapy provides numerous advantages in diagnostics and in drug delivery, especially for those malignant cells that are difficult to target or for drugs with poor bioavailability, such as those used for multiple myeloma (MM). This review summarizes the recent advances in the development of nanoparticle-based systems for the treatment of MM, taking into account the methods used for their functionalization, biocompatibility, and anticancer activity.

## 1. Introduction

Multiple myeloma (MM) is a malignant B-cell neoplasm characterized by the monoclonal plasma cells proliferation in the bone marrow (BM) [1]. This hematological cancer represents the second most common hematological malignancy after non-Hodgkin lymphoma. The World Health Organization (WHO) estimated in 2019 that 159,985 people are affected with multiple myeloma globally, with an overall 5-year survival rate of about 52% [2,3,4]. The excessive production of abnormal monoclonal immunoglobulin accumulated in the bone marrow stimulates osteoclastic activity, often leading to pathologic fractures [5]. Moreover, the elevated levels of these serum and and/or urine monoclonal paraproteins cause hematopoietic function failure and renal impairment [6].

The recent advances in genetic technologies have provided a better understanding of the molecular pathogenesis of MM, which is driven by initial somatic mutations and frequently associated with additional oncogenic mutations and interactions between the produced MM cells and their microenvironment [7]. So far, the treatment of this type of cancer relies on combined therapies using proteasome inhibitors [8,9], bisphosphonates as anti-resorptive agents for osteolytic bone lesions [10], corticosteroids [11], immunomodulatory drugs [12], and peripheral blood stem cell (PBSC) transplantation [13]. The current strategy of triplet therapy, followed by high-dose chemotherapy and autologous hematopoietic stem-cell transplantation, and finally by maintenance strategies led to improvements in overall survivals with a minimal residual disease (MRD) state [14]. However, despite the increased knowledge on the pathogenesis of MM and the development of new and effective therapeutic strategies, patients still have a worse prognosis as compared to most of the other hematological malignancies [15]. The disadvantages of conventional chemotherapy for MM are mainly related to the lack of effective cell targeting, systemic toxicity, adverse effects, and low therapeutic index. Moreover, most of the used anticancer agents for MM treatment show poor solubility in water-based solvents [16].

Nanomedicine has shown the potential to overcome these limitations through the development of advanced drug delivery systems (DDS) that are able to be transported into the complex bone marrow microenvironment (BMM) and to effectively reach myeloma cells. Several nanoparticle-based systems originating from organic or inorganic sources have been developed for a more effective and controlled drug release, prolonged blood circulation, and reduced toxicity [17]. When compared to small drugs or antibody–drug conjugates, nanoparticles offer new therapeutic possibilities due to their extraordinary chemical and physical properties, their high surface area to volume ratio that allows the incorporation of drug and/or imaging agents, their ability to augment tissue-specificity, and/or their ability to sustain controlled drug release [18]. It is noteworthy that nanosized drugs have shown increased accumulation in the tumor tissue, due to the leaky vasculature, as was observed for solid tumors. Considering that angiogenesis plays an important role in hematological malignancies including MM, nanoparticle-based drugs have the potential to serve as smart DDS, able to promote extravasation and retention phenomena in various compartments of BM, thus exerting a long-lasting therapeutic effect [19]. Moreover, nanoparticle-based diagnostic agents have shown great promise for translational clinical applications, being able to recognize a wide range of cell targets and thus enabling the evaluation and progression of MM [18,20,21]. In this review, we summarize the recent advances, published in the last five years, on the development of nanoparticle-based systems for the treatment of MM, taking into account the functionalization procedures, the biocompatibility, and the anticancer activity.

## 2. Nanotherapeutics for MM Therapy

Nanoparticle-based drug carriers and intrinsically active nanodrugs offer great potential in MM treatment enhancing treatment efficacy and reducing the side effects by acting preferentially in to the bone marrow niche in which myeloma cells develop. The delivery of anticancer drugs using nanoparticles can be achieved by different ways, which can be classified as passive, active, or triggered targeting [12,18]. The passive targeting approach takes advantage of the passive diffusion of nanoparticles through the tumor vascular endothelial barrier by an enhanced permeability and retention (EPR) effect, and it is particularly used for nanoparticles with longer blood circulation time, such as liposomes [22]. The active targeting involves the conjugation of nanoparticles with high-affinity ligands that can be selectively recognized by surface receptors on cancer cells, triggering receptor-mediated endocytosis; or surface receptors present on endothelial cells in order to minimize the systemic toxicity of conventional drugs [23]. Finally, nanopharmaceuticals have been prepared for the targeted release of drugs into the tumor site, which can be triggered by responsive mechanisms, such as pH, temperature, magnetic field, ultrasound, or light [24,25,26,27].

**Figure 1:**
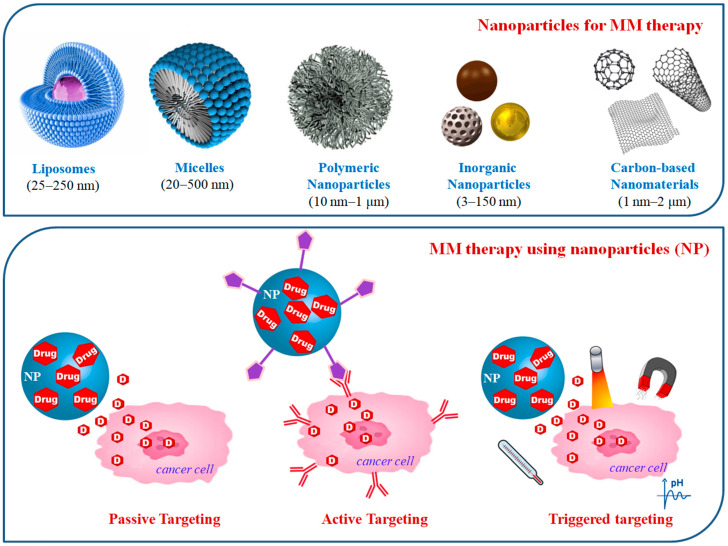
Nanodelivery approaches for multiple myeloma (MM) treatment.

The above reported different therapeutic approaches have been investigated using different classes of nanoparticles originating from organic or inorganic sources such as liposomes, micelles, and polymers, inorganic nanoparticles, and carbon-based nanomaterials—all with different shapes, sizes, and degrees of surface functionalization. For this purpose, different polymers were developed to formulate drug delivery systems approved as safe by the US-FDA and EU regulatory agencies. Poly(lactic-co-glycolic) acid (PLGA), polyethylene glycol (PEG), chitosan, poly-(ethylene oxide)-block-poly-(α-benzyl carboxylate ε-caprolactone; PEO-b-PBCL), and poly(ethylene imine (PEI) have gathered considerable attention due to their favorable properties. Some of the most attractive characteristics are their biodegradability and biocompatibility; the possibility to be combined with both hydrophilic and hydrophobic small molecules or macromolecules; efficient protection against degradation; sustained drug release; the possibility of surface functionalization to improve interaction with biological materials; and the ability to target specific organs, cells, or proteins [28]. The recent advances in the use of nanopharmaceutics for MM treatment (Figure 1), which have been classified for the different classes of the investigated nanoparticles, are reported in the following paragraphs and summarized in Table 1.

### 2.1. Liposomes

Liposomes (or artificial lipid vesicles) were the first nanoscale drug to be approved for clinical use in 1995. These small vesicles are frequently used due to their biocompatibility, non-immunogenicity, and their ability to enhance the water solubility of chemotherapeutic agents. In the last few years, some major breakthroughs in liposome technology have fueled the rapid development of new pharmaceutical liposomal applications [53]. Among the liposome drug delivery vehicles, PEGylated liposomal nanoparticles display high drug-loading capabilities, easy incorporation of different functionalities, elevated biocompatibility, and precise control over particle size. Carfilzomib (CFZ), a second-generation proteasome inhibitor, was loaded into liposomes with doxorubicin to enhance their anticancer effects at their optimal synergistic ratio. This combination with this specific delivery method demonstrated high stability and repeatability and an improved efficacy as compared to the free drug combination [22].

MM patients expressing VLA-4 (Very Late Antigen-4, also known as α4β1 integrin) and LPAM-1 (Leukocyte Peyer’s Patch Adhesion Molecule-1, also known as α4β7 integrin) display poor survival outcomes; thus, these targets can be used to develop new treatment strategies [29,54]. Since VLA-4 and LPAM-1 are ubiquitously expressed in healthy tissue, a dual-receptor targeted approach could be more effective in improving cell targeting and selectivity [55], as demonstrated in different cancer cell lines where binding selectivity was achieved by optimizing the valency. Using this strategy, a single targeting ligand that was not sufficient for effective targeting has been improved. Moreover dual-receptor targeted liposomes showed very low binding to cells displaying only one or none of the VLA-4 or LPAM-1 receptors, thus discriminating between myeloma and healthy cells. This approach demonstrated that the dual-receptor targeted strategy is extremely important to improve the selectivity and efficiency of targeted approaches, allowing therapeutic results that are otherwise unachievable with classical single targeting strategies [56].

A novel promising therapeutic target is BCMA (B-cell maturation antigen), which is a member of the tumor necrosis factor (TNF) receptor superfamily, together with the receptor for binding of B-cell activating factor (BAFF) and the proliferation-inducing ligand (APRIL) [57]. BCMA is prevalently expressed on MM and plasma cells, and it promotes cancer cell growth, survival, and drug resistance. For this reason, BCMA has being targeted by monoclonal antibodies, immunotoxins, bispecific T-cell engagers, and adoptive immunotherapy (e.g., CART, Chimeric Antigen Receptor T Cells). Two different nanoparticles formulations (PLGA- and lipid-based nanoparticles) encapsulating immunogenic heteroclitic BCMA_72−80_ [YLMFLLRKI] peptide have been tested to induce myeloma-specific CD8^+^ cytotoxic T lymphocytes. Both formulations displayed a similar rate of BCMA peptide encapsulation and showed optimal delivery to dendritic cells. The level of peptide loading was analyzed in a time-dependent manner; PLGA/peptide loading time was 18 h, while liposome/peptide achieved the maximum loading in only 30 min. Despite this difference, the PLGA/peptide induced a more effective BCMA-specific cytotoxic effect through CD107a degranulation-based cytotoxicity and the release of specific Th1-type cytokines. Thus, it is possible to speculate that a more efficient induction of cytotoxicity can be easily obtained with a “gradual” antigen uptake and the delivery of BCMA_72−80_ [YLMFLLRKI] [9,30].

Another promising strategy to treat MM is to develop novel formulations of glucocorticoids to improve their pharmacokinetic profile, reduce adverse effects, and enhance tumor accumulation. Fluorescently labeled liposomes loaded with dexamethasone (DEX) were found in plasma up to 24 h after parental administration in a human-mouse hybrid model of MM. Liposomes accumulated in cancer-bearing human-bone scaffolds and strongly reduced tumor growth, while free dexamethasone was inactive at an equivalent dose [31].

### 2.2. Micelles

Micelles are artificial vesicles similar to liposomes which are formed by the self-assembly of amphiphilic lipids enclosing a hydrophobic core, where hydrophobic drugs can be loaded. Polymeric micelles represent promising nanocarriers for cancer-targeted delivery due to their excellent biocompatibility, prolonged circulation time, suitable particle size (10–100 nm), and the ability to dissolve a broad variety of poorly soluble pharmaceuticals. This capability has led to the development of several types of drug-loaded micelles that are currently being tested in preclinical and clinical trials. Among polymeric micelles, a special group is formed by lipid-core micelles, i.e., micelles formed by conjugates of soluble copolymers with lipids, such as polyethyleneglycol phosphatidylethanolamine (PEG-PE) conjugates [58].

Carfilzomib (CFZ) was introduced on the market as a treatment option for the refractory MM, and many efforts have been made to increase its short circulation time. Recently, [32] an A6 peptide (KPSSPPEE)-tagged core-disulfide-crosslinked biodegradable micelle, named A6-polimeric micelle (A6-PMs), prepared from polyethyleneglycol-b-polydithiolane trimethylene-co-ε-caprolactone (PEG-P(DTC-*co*-CL)) copolymers was designed to obtain CFZ delivery in human MM cell lines, LP-1, overexpressing CD44-receptors. A6 is a little octapeptide of 40 nm, with a good antiangiogenic activity in mice and stable CFZ loading, high stability, ability to target CD44, and a lower systemic toxicity with respect to other CFZ-sulfobutylether-β-cyclodextrin (CFZ-CD), which, on the contrary, showed a short half-life, absence of selectivity, and poor anticancer activity.

Since CFZ is a proteasome inhibitor with chymotrypsin-like activity (β5c subunit), the inhibitory profile of several CFZ preparations in LP-1 cell lines was evaluated. The results of this investigation clarified that both CFZ-A6-PMs and CFZ-PMs showed a significant rate of inhibition (i.e., 85% and 80%, respectively) after an incubation of 1 h with LP-1 cells, while CFZ-CD showed a lower inhibition rate (59%). In addition, confocal laser scanning showed that cells treated with A6-PMs-Cy5, where Cy5 is fluorescein cyanine 5 (a fluorescent dye), showed a more intense cytosol fluorescence than PMs-Cy5, thus revealing that the A6 peptide enhances the endocytosis process due to its high affinity to CD44.

Varela-Moreira et al. synthesized new CFZ-PM based on poly(ethylene glycol)-b-poly(*n*-2-benzoyloxypropyl methacrylamide, which is also known as mPEG-b-p(HPMA-Bz), with the aim to increase the highest tolerated dosage of CFZ in a humanized bone marrow-like scaffold (huBMsc) xenograft model, which is a human–mouse hybrid animal model with many features of human MM disease. Release in this case occurs by means of albumin, which possesses lipophilic pockets for the accommodation of lipophilic drugs [33]. These types of micelles showed a 55 nm size for extended circulation after parenteral injection. The effect on cell viability of MM1.S, L363, and UM-9 cell lines was investigated for CFZ-PM, CFZ complexed in sulfo-butylether-β-cyclodextrin (CFZ-CD), and free CFZ. The IC_50_s of CFZ-PM in MM1.S, L363, and UM-9 cells were of 5.3, 6.8, and 6.1 nM, respectively, while for CFZ-CD and free CFZ, the obtained IC_50_ were improved, and the reduced cytotoxic effect of the CFZ-PM formulation can be due to CFZ retention in the PM core. The tolerability of CFZ-PM was tested in the huBMsc model xenografted with MM1.S cells in comparison to CFZ-CD by monitoring mice body weight. A weight loss inferior to 5% was observed within the first week; compared with CFZ-CD, it showed a reduction in weight loss of 20%. These results clearly suggested that the tolerability of CFZ-PM was definitely improved with respect to that of CFZ-CD, thus indicating that higher doses/or frequent doses of CFZ-PM could be safely administered to mice. Despite CFZ-PM not showing toxicity in treated mice, there was a lack of antitumor activity (in terms of tumor size) with respect to CFZ-CD in bioluminescence imaging analysis.

Another interesting study has been carried out on bortezomib (BTZ), which is a first-generation proteasome inhibitor, which despite representing the first-line therapy of MM shows fast clearance, low selectivity, and a relevant toxicity as peripheral neuropathy and myelosuppression. Gu et al. produced novel hyaluronic acid-shelled and core-disulfide-crosslinked biodegradable micelles (HA-CCMs) loading BTZ or bortezomib–pinanediol (BP) [34]. The new micelles exhibited a 78 nm size, excellent stability, and proved to release the drug upon adding GSH. The new HA-CCMs containing BP showed in MM LP-1 cell lines over-expressing CD44-receptors an improved suppression of tumor growth with respect to the free BP in LP-1 MM tumor-bearing mice.

On the contrary, HA-CCMs-BP showed a comparable inhibition of proteasome with respect to the free BTZ. Cy5-labeled HA-CCMs proved that HA-CCMs have a prolonged half-life and an increased tumor retention due to an HA-mediated mechanism of uptake. These recent examples of new polymeric micelles capable of encapsulating potent proteasome inhibitors show that it is possible to increase the half-life and selectivity of chemotherapeutics, reduce toxicity, and improve the antitumor response.

Another promising strategy to treat MM is to transform chemotherapeutics to photoactivatable drugs using radiopharmaceuticals [35]. More in detail, novel nanomicells (NM) were synthesized to realize a selective delivery in MM cells and at the same time a co-localization of radiolabeled fluorodeoxyglucose (^18^FDG) and titanocene in MM cells, leading to the selective death of cancer cells with a good selectivity profile over normal stem cells. Titanocene (TC) was used as a photosensitizer because of its UV light excitability and easy biodegradability. It was demonstrated that NM-TC enter the bone marrow and bind to α4β1 receptor on tumor cells delivering the drug to the cells; 2 h after ^18^FDG is administered, it enters the cancer cells through the overexpressed Glut transporters. Once the drug and ^18^FDG are localized in the tumor cells, the former is photoactivated by the latter through Cerenkov radiation, leading to selective tumor cell death [35].

### 2.3. Polymeric Nanoparticles

In addition to micelles, copolymers can also form nanoparticles. However, the equilibrium dynamics of nanoparticles is different from that of micelles as mentioned earlier. The micellization of a copolymer is a spontaneous process based on the self-assembly and thermodynamic aspects of copolymer monomers, while the formation of nanoparticles is “kinetically” controllable by several factors, such as temperature, pH level, electrolytes, solvent contents, etc., and proceeds through a fast addition of monomers and a subsequent slow rearrangement. The colloidal stability of these nanoparticles is maintained by steric or electrostatic repulsion preventing fusion from Brownian collision [59]. Therefore, polymeric nanoparticles (PNPs) are particles prepared from natural or synthetic biodegradable or non-biodegradable polymers, where the therapeutic agent is dissolved, adsorbed, entrapped, or encapsulated into the matrix. Hybrid PNPs, constituted by chitosan and poly(lactic-co-glycolic acid) (PLGA), have been developed to encapsulate miR-34a, which is a well-known tumor suppressor miRNA that has proven to be a potential therapeutic candidate in the treatment of MM [36]. These PNPs proved to be able to efficiently protect genetic material from degradation by serum enzymes such as ribonuclease. Cosco et al. reported a significant in vitro antitumor effect against MM cells, while the evaluation of the therapeutic properties in vivo, against human MM xenografts in NOD-SCID mice revealed an inhibition of tumor growth through systemic injection and increased mice survival.

**Figure 2:**
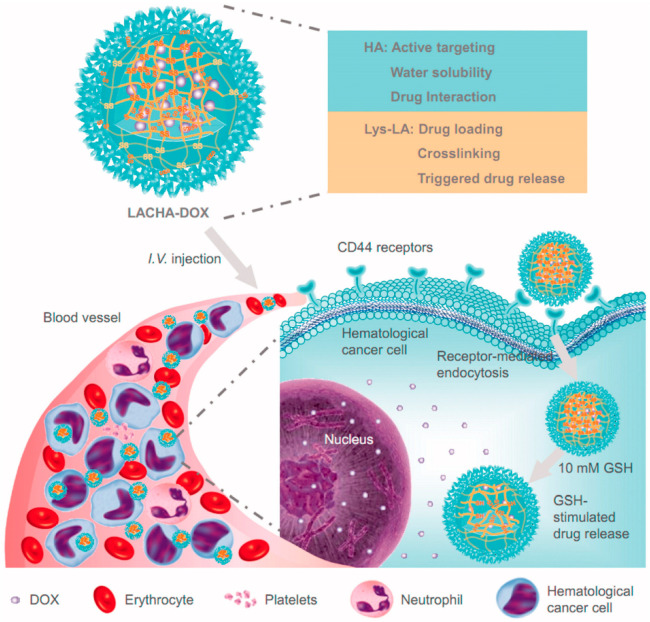
Doxorubicin (DOX)-encapsulated lipoic acid-crosslinked hyaluronic acid nanoparticles (LACHA) for the treatment of CD44 over-expressed hematologic malignancy. Reproduced from Ref. [37].

Zhong et al. reported the synthesis of lipoic acid-crosslinked hyaluronic acid nanoparticles (LACHA-DOX) loaded with doxorubicin for the targeted inhibition of LP-1 human MM and AML-2 (human acute myeloid leukemia cells) xenografted in nude mice [37]. The authors used hyaluronic acid (HA) as a targeting ligand that possesses an intrinsic binding affinity to over-expressed CD44 receptors in many tumor cells (Figure 2).

The LACHA-DOX nanoparticles showed a high in vitro targeting ability and anticancer activity toward over-expressing CD44 receptor cancer cells. The in vivo and ex vivo studies reported an increased accumulation of PNPs in LP-1 and AML-2 tumor xenografts. Mice were treated with the drug-loaded system or with the free DOX when tumors grew up to about 80 mm^3^ in volume.

Encapsulated DOX reduced tumor size in both groups, and when compared to controls, the treated mice showed a prolonged survival. Encapsulated DOX caused little damage to major organs such as the liver and heart. This study suggests that the targeting approach using HA may offer a valuable tool for the treatment of CD44^+^ cancer cells. Karri et al. developed PLGA-NPs for the release of Lenalidomide (LND), which is an anticancer drug used in MM therapy, in order to improve its bioavailability and to achieve a sustained release [38]. In vitro release studies of the LND-loaded PNPs showed a biphasic pattern of drug release. About 40% of drug was released within 6 h, while amounts of 77% and 83% of drug were released at 24 and 48 h, respectively.

The relative bioavailability was increased about 3.67-fold compared to free LND after oral administration in Wistar rats, thus demonstrating the great potential of the polymeric drug delivery system for MM therapy.

An example of PNPs-mediated targeted delivery of proteasome inhibitor bortezomib (BTZ) was reported by de la Puente et al. [39]. To reduce the side effects in healthy tissue, the authors loaded BTZ in crosslinked chitosan NPs, which were conjugated with anti-CD38 monoclonal antibodies. Chitosan NPs have been obtained by ionic gelation crosslinking with sodium tripolyphosphate solution (TPP) and conjugated with streptavidin; then, the biotin–anti-CD38 was added to the system in order to obtain anti-CD38 targeted chitosan NPs. In vitro studies showed an increased cytotoxicity and apoptosis on MM cells when compared to the free BTZ. In vivo studies performed on tumor-bearing mice with vehicle demonstrated that the targeted BTZ-NPs were able to reduce tumor size when compared to the non-targeted NPs and to the free drug.

Lee et al. reported an injectable coacervate hydrogel for MM therapy by encapsulating BTZ in micellar nanoparticles possessing a catechol-functionalized polycarbonate core through a pH-sensitive covalent bond between the biodegradable phenylboronic acid (PBA) in BTZ and catechol [40]. The drug-loaded micelles were obtained through ionic coacervation between PBA–cationic guanidinium- or thiouronium-functionalized polycarbonate (“A” block) and poly(ethylene glycol) (PEG) (“B” block). The “ABA” triblock copolymer was synthesized through organocatalytic ring-opening polymerization (OROP) starting from PEG with double ended diols and using PBA as a crosslinker agent. In vitro release studies performed on the BTZ-loaded NPs showed the pH dependence of drug release; the BTZ release from the micelle/hydrogel composite remained at 7% at pH 7.4, whereas in an acidic environment, around 85% of BTZ was released gradually over 9 days. The results of in vivo studies performed in an MM.1S xenograft mouse model showed a similar tumor progression in mice treated with BTZ-loaded micelles when compared to the control group, whereas the treatment with the drug-loaded micelle/hydrogel composite showed a significant delay in the tumor progression, thus demonstrating the potential of the hydrogel for subcutaneous administration and sustained drug delivery.

Huang et al. have also reported the development of monoclonal anti-CD38 antibody–conjugated NPs in MM therapy [41] using NPs loaded with the S3I-1757 inhibitor of the STAT3, which is an oncoprotein that has proved to induce drug resistance in MM. In this study, the first unmarked NPs were labeled as S3I-NP, while the NPs marked with anti-CD38 were labeled as CD38-S3I-NP. To generate CD38-S3I-NP, anti-CD38 was first thiolated and then combined with maleimide-functionalized poly(ethyleneoxide)-block-poly(-benzylcarboxylate-”-caprolactone) (PEO-b-PBCL) and finally linked to the polymers. The anti-CD38-conjugated polymers were mixed with NPs and loaded with S3I-1757 (S3I-NP) to generate the CD38-S3I-NP and S3I-NP nanosystems. Drug release studies performed with the two formulations showed a comparable drug release (about 68%). Moreover, the authors conjugated the fluorophore Cy5.5 to NPs with or without anti-CD38 coating (named as Cy5.5-CD38-NP and Cy5.5-NP, respectively) and reported a significant increase of Cy5.5-CD38-NP uptake in two MM cell lines (U266 and RPMI8226). CD38-S3I-NP showed a lower inhibitory concentration (IC_50_) with respect to S3I-NP in IL6-stimulated MM cells. In vivo studies performed on a xenograft mouse model demonstrated that CD38-S3I-NP was able to significantly reduce the tumor size by 4-fold when compared to S3I-NP, 12 days after drug administration. The ability of CD38-S3I-NP to suppress the STAT3 phosphorylation was also proved using Western blot analysis and immunocytochemistry. Bae et al. developed BCMA-specific peptide encapsulated PLGA nanoparticles to trigger BCMA-specific CD8^+^ cytotoxic T lymphocytes endowed with immune activities against MM. [30] The heteroclitic BCMA_72−80_ [YLMFLLRKI] NPs have been synthesized by a double emulsion-solvent technique using polyvinyl alcohol (PVA) to stabilize emulsions and showed uniform size distribution and increased peptide delivery to human dendritic cells, thus enhancing BCMA-specific CTL induction. The authors reported a gradual increase in peptide uptake by antigen-presenting cells as evaluated by flow cytometry analysis and higher anticancer activities against primary CD138^+^ tumor cells and MM cell lines. The results of this study underlined the potential of the PLGA-based BCMA immunogenic peptide delivery systems, instead of the free peptide, to enhance the induction of BCMA-specific CTL through polyfunctional Th1-specific activities against MM and also suggested the potential clinical utility of PLGA-based cancer vaccines to improve the BCMA-targeted immunotherapy against MM.

Guo et al. have recently developed a DNA vaccine containing the cancer-specific antigen Dickkopf-1 (DKK-1) and the programmed death-ligand 1 (PD-L1), delivered by PLGA/PEI NPs, for MM therapy [42]. In this study, mice subcutaneously inoculated with over-expressing hDKK-1-Sp2/0 cells were immunized with the vaccine PLGA/PEI-pPD-L1/pDDK-1 and equal amounts of control. The PLGA/PEI-pPD-L1/pDKK-1 co-immunization increased the immunogenicity of DKK-1 antigen and inhibited tumor growth. Moreover, the anticancer activity of the synthesized vaccine was required for CD8^+^ and CD11c^+^ DCs-mediated CD8^+^ T cell response, thus highlighting the potential of this vaccine strategy as a promising approach for hematological malignancy treatment.

### 2.4. Inorganic Nanoparticles

Advanced drug delivery systems, bio-imaging, and diagnostic tools based on inorganic nanoparticles have been widely investigated for MM therapy [17,18]. Gold and metal oxide nanoparticles such as iron or zinc oxide and mesoporous silica nanoparticles show the advantage of being synthesized through simple approaches with high purity, near monodispersity, strictly controlled physicochemical and morphological characteristics, and with tailored surface functionalization [60]. Due to their high surface area and pore volume and their ease of surface modification, the mesoporous silica nanoparticles (MSNs) received great interest in nanomedicine in recent years [61]. MSNs have been synthesized with controllable size (30–200 nm) for tumor targeting via enhanced permeation and EPR effect [62] and have shown the ability to act as acid-responsive nanocarriers for targeted anticancer drug delivery [63]. Surface-engineered mesoporous silica luminescent nanoparticles decorated with cancer targeting ligands have also shown the potential for use for cancer targeting and bio-imaging applications [64,65]. Snake venom-loaded silica nanoparticles with a particle size of approximately 300 nm have been investigated as anticancer agents for MM cancer cells both in vitro and in vivo [43]. Iodinated silica/porphyrin hybrid nanoparticles have been developed to enhance the therapeutic efficacy by combining photodynamic and photothermal therapy [66]. These nanoparticles have shown the ability to generate ^1^O_2_ and heat in the human myeloma cell line RPMI 8226 after irradiation by a light-emitting diode (LED), thus acting as photosensitizers in combination therapies. Moreover, the decoration of these nanosystems with the targeting ligand folic acid allowed the preferential accumulation in the tumor area 18 h after intravenous injection in tumor-bearing mice. The LED irradiation caused an inhibition of tumor growth and improvement in the survival rate.

Gold nanoparticles have long proved their potential as therapeutic tools for the treatment of MM, acting as inhibitors of the MM cell line proliferation by cell-cycle arrest in the G1 phase via the up-regulation of cyclin-dependent kinases inhibitors, p21 and p27 [67]. Bare gold nanoparticles have also shown the ability to arrest the S phase; this effect is of particular interest for slow-growing malignancies such as MM, where a great portion of cells remain in the G1 phase, showing also the possibility of targeted drug delivery [68]. Moreover, gold nanorods have shown to be effective contrast agents for detecting single myeloma cells in blood circulation using speckle-modulating optical coherence tomography (OCT) within blood in vivo [69]. A tetrahedral bis-chelated gold(I) phosphine complex [Au(d2pype)_2_]Cl was recently investigated as an inhibitor of thioredoxin reductase (TrxR) in bortezomib-sensitive or resistant myeloma cells [44]. The complex proved to significantly reduce cell proliferation and to induce apoptosis, both of which were found to be reactive oxygen species (ROS)-dependent. The authors also demonstrated a significant anti-MM activity in vivo in a human RPMI8226 xenograft model of immunocompromised NOD/SCID mice.

Magnetic iron oxide nanoparticles have raised great attention in nanomedicine due to their biocompatibility, biodegradability, and low toxicity [70]. Shi et al. demonstrated the ability of ferroferric oxide nanoparticles (Fe_3_O_4_) to induce in blood cells autophagy, which showed to protect MM cells from apoptosis induced by anticancer agents [71]. Magnetic *γ-*Fe_2_O_3_ nanoparticles with hydrodynamic sizes from 70 to 75 nm, capped with dimercaptosuccinic acid and conjugated with the anticancer drug paclitaxel and the anti-ABCG2 monoclonal antibody, were developed in order to evaluate the combined therapeutic efficacy toward RPMI 8226 by evaluating the negative expression of CD138 and CD34 [45]. The results of in vitro and in vivo studies demonstrated the ability of the nanosystem to inhibit the growth of CD138^−^ and CD34^−^ cells as well as of their related tumors in xenografts. This effect was correlated to the increased expression of caspase-9, caspase-8, and caspase-3, and the down-regulation of NF-kB, thus offering a valuable approach to MM treatment through an apoptotic pathway. Fe_3_O_4_ magnetic nanoparticles modified with dimercaptosuccinic acid, with a size of 26 nm, have been also conjugated with nontoxic concentration of bortezomib and gambogic acid (GA), and their anticancer activity was tested on RPMI-8226 cells [46]. The authors demonstrated the ability of the nanosystem to increase G2/M phase cell cycle arrest and to induce apoptosis in vitro, phosphorylating Akt, thus increasing caspase-3 and Bax expression, and down-regulating PI3K and Bcl-2. The in vivo studies performed on a tumor xenograft model in male BALB/c nude mice indicated that the system was also able to decrease tumor growth and volume.

Arsenic trioxide (As_2_O_3_) in combination with bortezomib, melphalan, and ascorbic acid have shown efficacy in a phase II randomized clinical trial in patients with relapsed/refractory MM [72]. The cytotoxic activity of As_2_O_3_ against a panel of MM cell lines and CD138+ MM cells was compared against realgar (As_4_S_4_) nanoparticles [47]. In this study, As_4_S_4_ nanoparticles with a mean particle size of 131 nm showed less genotoxicity and good tolerability when compared to As_2_O_3_. In vivo experiments performed in MM patient-derived huBMsc mouse models showed anti-MM activity for both compounds, resulting in a significant reduction of tumour burden. As_4_S_4_ nanoparticles induced G2/M cell cycle arrest and the modulation of cyclin B1, p53 (TP53), p21 (CDNK1A), Puma (BBC3), and Wee-1 (WEE1). Notably, As_4_S_4_ nanoparticles greatly reduced the proportion and clonogenicity of the MM stem-like side cells, as also observed in co-culture with bone marrow stromal cells. Moreover, the authors reported a synergistic effect when both compounds were combined with the anti-MM drugs lenalidomide or melphalan.

Zinc oxide (ZnO) nanoparticles have also proven their potential use as drug nanocarriers reducing toxicity and side effects [73]. The influence of ZnO nanoparticles on RPMI-8226 was recently investigated in vitro by Xu et al. [48]. In this study, ZnO nanoparticles (average diameter of about 30 nm) efficiently induced MM cell death in a dose- and time-dependent manner. The authors reported that the main pathways activated by ZnO nanoparticles include oxidative stress-induced mitochondrial dysfunction and caspase-dependent death.

Among the most recent advances in theranostics, Tang et al. developed novel titanium dioxide nanoparticles that are able to generate reactive oxygen and coated with transferrin and radiolabeled with ^89^Zr as a radionuclide capable of targeting the bone marrow. This strategy allows the visualization of nanoparticle distribution and the generation of reactive oxygen species (ROS) that trigger the apoptotic pathway [49]. Studies on tissue biodistribution carried out by means of positron emission tomography or X-ray computer tomography suggested that the administration of ^89^Zr-radiolabeled nanoparticles takes advantages of the ^89^Zr osteotropic effect for the selective localization in mouse bone tissue.

### 2.5. Carbon-Based Nanoparticles

Among the different classes of nanomaterials, the carbon-based materials characterized by a graphene structure, such as graphene oxide (GO), carbon nanotubes (CNTs), fullerenes, and graphene quantum dots (GQDs) have received significant interest in anticancer therapy due to their small size (normally in the range of 1–100 nm) and unique chemical and physical properties [74,75,76,77]. However, very little is known about the cytotoxicity of graphene-based materials and their anticancer activity on hematological tumor cells. The cytotoxic effects of GO and of doxorubicin (DOX)-conjugated GO were evaluated on human MM cells by Wu et al. [50]. RPMI-8226 cells were treated with DOX, GO, and with the conjugated system GO/DOX, and cell viability was evaluated by analyzing cell cycle and apoptosis. The results obtained from this study underlined the low cytotoxicity of GO and its ability to release DOX to cancer cells, since GO/DOX significantly inhibited MM cell proliferation as compared to the unconjugated drug. In a different work, the same research group evaluated the toxicity of GO and its possible mechanisms by using RPMI 8226 cells [78]. The authors found that GO induced cytotoxicity in a dose-dependent manner and showed low cytotoxicity at concentrations lower than 100 mg/L. Moreover, it was reported that GO did not induce apoptosis and that the primary cytotoxic mechanism caused by this nanomaterial was due to the generation of oxidative stress.

In a recent study, Lin et al. conjugated single-walled carbon nanotubes (SWCNTs) with a metastasis-associated lung adenocarcinoma transcript 1 (MALAT1), which represents a highly conserved lncRNA (long non-coding RNA) able to regulate DNA repair and MM cell death [51]. In this work, the authors functionalized SWCNTs with PEG-2000 to improve its water solubility; thus, it was covalently conjugated to the nanosystem and the thiolated anti-MALAT1 gapmer DNA. The in vitro studies on H929 and MM.1 S cells demonstrated that the anti-MALAT1 gapmer DNA was able to efficiently knock down the MALAT1 expression. When injected intravenously into a disseminated MM mouse model, a remarkable inhibition of MM progression was observed, thus indicating SWCNTs as ideal delivery shuttles for anti-MALAT1 gapmer DNA.

In another recent study, doxorubicin (DOX) was loaded in polyethylene glycol-modified cadmium telluride quantum dots (PEG-CdTe QDs) with the aim to promote drug accumulation in the intracellular environment and release DOX in a pH-controlled manner, thus improving the rate of apoptosis of RPMI-8226 through regulating the expression of apoptosis-associated genes [52].

## 3. Conclusions

The conventional therapy for the treatment of multiple myeloma suffers from severe limitations such as low targeting ability, poor bioavailability, systemic toxicity, and emerging drug resistance. Most anticancer drugs are often poorly soluble, requiring the use of excipients for an efficient use and adequate delivery to cancer cells. Nanotherapeutic formulations could represent an effective alternative strategy to improve drug release, prolong circulation in blood, increase payload in tumors, and minimize off-target toxicity. The unique properties of nanoparticles and their high surface area to volume ratio allow the incorporation of drug and/or imaging agents in the same nanoplatform. The possibility offered by the nanoparticles multi-functionalization can allow the co-delivery of different anticancer agents, thus further improving the chemotherapy efficacy of anticancer agents endowed with different pharmacological activities. The combination chemotherapy, widely used for MM treatment, could be dramatically improved by inserting two or more anticancer agents in the same nanoplatform, thus affording a synergistic enhancement of therapeutic efficacy while reducing side effects and drug resistance. Moreover, prolonged drug circulation in the bloodstream, reduced frequency of dosage and uniform, and sustained drug release kinetics could be achieved. However, despite the outstanding chemical, physical, and biological properties offered by nanosystems and the great promise envisaged by nanoparticles-mediated combination therapy, relevant pre-clinical models on different animals for testing their targeting efficiency and the potential long-term toxicity are required by using interdisciplinary approaches.

## Figures and Tables

**Table 1:** Nanotherapeutics for MM treatment.

Nanotechnology Platform/Anticancer Agent	Targeting Ligand	Trigger System	Imaging Studies	Biological Test	Ref.
PEG-liposomes/DOX/CFZ	-	-	-	MM.1S and NCI-H929 cells, SCID mice	[22]
PEG-liposomes	VLA-4 and LPAM-1	-	-	NCI-H929, MM.1S, U266, IM9, RPMI-8226 cells	[29]
BCMA-liposomes	BCMA-specific peptide	-	-	CD138^+^ cells	[30]
PEG-liposomes/DEX	-	-	in vivo	MM.1S cells, RAG2^−/−^γc^−/−^mice	[31]
PEG-P(DTC-*co*-CL) micelles/CFZ	A6 peptide (KPSSPPEE)	-	in vivo and ex vivo	LP-1 cells, LP-1 MM-bearing nude mice	[32]
PEG-b-p(HPMA-Bz) micelles/CFZ	-	-	in vivo	MM1.S, L363 and UM-9 cells, Female RAG2^−/−^γc^−/−^mice	[33]
HA-CCMs micelles/BTZ	HA	-	in vivo	LP-1 cells	[34]
TC micelles/FDG	VLA-4-		in vivo	MM1.S-Luc cells, C57BL/6J mice	[35]
chitosan-PLGA/miR-34a	-	-	-	SKMM1 and 8226 cells, SKMM1 xenograft models in SCID mice	[36]
LACHA/DOX	HA	-	in vivo and ex vivo	LP-1 and AML-2 cells, LP-1 and AML-2 tumor xenografts	[37]
PLGA/LND	-	-		U266 cells, Wistar rats	[38]
Chitosan/BTZ	biotin-anti-CD38	-	in vivo	MM.1S, H929, RPMI8226 and U266 cells, SCID mice	[39]
catechol-polycarbonate-PBA/BTZ	-	pH	-	MM.1S and HDF cells, MM.1S xenograft mouse	[40]
PEO-b-PBCL/S3I-1757	anti-CD38	-	in vivo and ex vivo	U266 and RPMI8226 cells, SCID xenograft mouse	[41]
PLGA	BCMA-specific peptide	-	-	CD138^+^ cells	[30]
PLGA-PEI -pPD-L1/pDDK-1	pDDK-1	-	-	splenocytes, female BALB/c mice	[42]
silica/porphyrin	FA	LED	in vivo	RPMI 8226 cells, female CB17/Icr-*Prkdc scid* mice	[43]
[Au(d2pype)_2_]Cl	-	-	in vivo	JJN3, RPMI8226 and U266 cells, NOD/SCID mice	[44]
γ-Fe_2_O_3_/paclitaxel	Anti-ABCG2	-	-	RPMI 8226 cells, male NOD/SCID mice	[45]
Fe_3_O_4_/BTZ/GA	-	-	-	RPMI-8226 cells, male BALB/c nude mice	[46]
As_4_S_4_/melphalan or lenalidomide	-	-	-	RPMI 8226-S, RPMI-Dox40, RPMI-LR5, RPMI-MR20, MM.1S, MM.1R, OPM-1, OPM-2, KMS-11, KMS-18, OCIMY5, U266, NCI-H929, HS-5 cells, huBMsc mouse models	[47]
ZnO	-	-	-	RPMI8226 cells	[48]
^89^Zr radiolabeled TiO_2_	transferrin	-	in vivo	MM1.S, SCID mices	[49]
GO/DOX	-	-	-	RPMI8226 cells	[50]
SWCNTs	anti-MALAT1 gapmer DNA	-	in vivo	H929, MM.1 S cells, SCID-beige mouse	[51]
PEG-CdTe/DOX		–pH	-	PRMI8226 cells	[52]
